# De novo gonad transcriptome analysis of the common littoral shrimp *Palaemon serratus*: novel insights into sex-related genes

**DOI:** 10.1186/s12864-019-6157-4

**Published:** 2019-10-22

**Authors:** Inés González-Castellano, Chiara Manfrin, Alberto Pallavicini, Andrés Martínez-Lage

**Affiliations:** 1 Universidade da Coruña, Departamento de Biología and Centro de Investigaciones Científicas Avanzadas (CICA), 15071 A Coruña, Spain; 2 Università degli Studi di Trieste, Dipartimento di Scienze della Vita, 34127 Trieste, Italy

**Keywords:** Shrimp, Sex-related, Ovary, Testis, RNA-Seq, Transcriptome, DEG, Aquaculture

## Abstract

**Background:**

The common littoral shrimp *Palaemon serratus* is an economically important decapod resource in some European communities. Aquaculture practices prevent the genetic deterioration of wild stocks caused by overfishing and at the same time enhance the production. The biotechnological manipulation of sex-related genes has the proved potential to improve the aquaculture production but the scarcity of genomic data about *P. serratus* hinders these applications. RNA-Seq analysis has been performed on ovary and testis samples to generate a reference gonadal transcriptome. Differential expression analyses were conducted between three ovary and three testis samples sequenced by Illumina HiSeq 4000 PE100 to reveal sex-related genes with sex-biased or sex-specific expression patterns.

**Results:**

A total of 224.5 and 281.1 million paired-end reads were produced from ovary and testis samples, respectively. De novo assembly of ovary and testis trimmed reads yielded a transcriptome with 39,186 transcripts. The 29.57% of the transcriptome retrieved at least one annotation and 11,087 differentially expressed genes (DEGs) were detected between ovary and testis replicates. Six thousand two hundred seven genes were up-regulated in ovaries meanwhile 4880 genes were up-regulated in testes. Candidate genes to be involved in sexual development and gonadal development processes were retrieved from the transcriptome. These sex-related genes were discussed taking into account whether they were up-regulated in ovary, up-regulated in testis or not differentially expressed between gonads and in the framework of previous findings in other crustacean species.

**Conclusions:**

This is the first transcriptome analysis of *P. serratus* gonads using RNA-Seq technology. Interesting findings about sex-related genes from an evolutionary perspective (such as Dmrt1) and for putative future aquaculture applications (Iag or vitellogenesis genes) are reported here. We provide a valuable dataset that will facilitate further research into the reproductive biology of this shrimp.

## Background

The common littoral shrimp *Palaemon serratus* (Pennant, 1777) is a crustacean decapod with a geographical distribution ranging the Atlantic Ocean (from Scotland and Denmark to Mauritania, including Azores, Madeira and Canary Islands) and the entire Mediterranean Sea and the Black Sea [1]. This species inhabits the intertidal and subtidal soft-sediment of estuaries in the reproductive season, and rocky bottoms covered with seagrass and algae [2, 3]. *Palaemon serratus* fishing activity is crucial in some European communities, mainly around the British Isles, France and northern Spain [4]. In Galicia (NW Spain) the volume of catches varies from 47.6 to 90.7 tons traded per year, what equals to worth of 2 million euros per year in this region (data obtained from https://www.pescadegalicia.gal/ on 26 Sep 2018, Xunta de Galicia). The high commercial value of this species could possibly lead to overfishing [4]. Implementation of proper management measures that ensure a sustainable exploitation will prevent depletion or genetic deterioration of wild fisheries [5, 6]. Aquaculture practices might improve *P. serratus* production at once reduce the fishing pressure over the wild populations.

In the field of aquaculture, reproductive traits are considered economically significant. Hence, understanding sexual and reproductive development is necessary to obtain successful and sustainable cultures, to increase seed quality or to breed genetically improved lines [7, 8]. For instance, as sex dimorphism in growth is common in crustaceans, monosex aquaculture of commercially relevant species is especially interesting. In monosex populations the yield is increased because energy from reproduction is invested in growth, resulting in larger-size individuals than in sex-mixed cultures [9, 10]. A better knowledge about the genetics of crustacean sexual development facilitates the application of biotechnological strategies, such as sex-change induction, benefiting productivity [7].

Sexual development includes sex determination and sex differentiation processes. In Decapoda, sex is determined by the initiation of a genetic cascade triggered by a master sex-determining gene. Downstream genes in this cascade act as sex-regulator genes, leading to the sex differentiation pathway, which in turn results in a sex-specific phenotype development [11]. Due to the lack of genomic information in crustacean decapods, sex-determining genes have not been identified and even sex-related genes have been rarely reported [9]. Several genes are considered preliminary candidates to be implicated in decapod sex determination but since these genes have been identified through homology screening, the list is heavily biased by genes characterized in model species as *Drosophila melanogaster*, *Caenorhabditis elegans* and Mammalia (e.g. Sxl, Tra, Tra-2, Dsx, Fem-1 or Sry; see review in [11]). Among these candidates, it is noteworthy to highlight that Dmrt genes (doublesex and male abnormal-3-related transcription factors) have been noted as the only gene family with a conserved function in sex determination across metazoans [11, 12], so they are particularly intriguing. About sex differentiation, the insulin-like androgenic factor (IAG) is a well characterized hormone with a conserved central role in Malacostraca. Iag expression in the androgenic gland (AG) leads sexual differentiation to maleness by governing the onset of testicular and secondary-sex characteristics development in males. Upstream in the sex differentiation pathway, an array of neuropeptides secreted by the eyestalk regulates the Iag expression [11]. It was shown that AG-implanted females become males, and conversely AG-ablated males turned into females (see review in [13]). As this surgical procedure implies a high mortality rate, it has been recently achieved that Iag-silenced males shifted phenotypically into females in the prawn *Macrobrachium rosenbergii* [14]. Thus, the biotechnological manipulation of the expression of sex determination or sex differentiation genes has the potential to improve the aquaculture production, such as creating monosex populations among other possibilities. Nevertheless, prior to the implementation of any genetic manipulation technique, it is necessary a depth-understanding about the genetic factors underlying the sexual development in *P. serratus.*

RNA-Seq greatly enhances the capability for gene discovery in non-model organisms where genomic data is not available. Transcriptome profiling using high-throughput sequencing allows the identification of transcripts involved in biological processes [15]. Comparative transcriptomics and differential expression analyses (DEA) between female and male reproductive tissues enable the detection of transcripts with sex-biased and sex-specific expression. In fact, sex determination and sex differentiation genes have been identified in several commercial decapod species thanks to transcriptomic analyses of certain tissues, including gonads [9, 16–18].

Genetic studies in *P. serratus* are scarce and mainly focused in its population genetics and cytogenetics. Solely one transcriptomic work is available for this species, providing data to study larval development and metamorphosis [19]. Regarding to sex determination in *P. serratus,* it is only known that heteromorphic sex chromosomes are absent [20]. No sex determination or sex differentiation genes or pathways have been reported for this shrimp. Accordingly, the aim of the present study was to identify candidate genes to be involved in the sexual development of *P. serratus*. For this purpose, an ovary and testis transcriptome was assembled and annotated from Illumina high-throughput sequencing reads, and genes with differential expression between ovarian and testicular tissues were studied. To the best of our knowledge, this is the first work that address the transcriptome profile analysis of both male and female *P. serratus* gonads, providing new data about sex-related genes in this species.

## Results

### Quality control, trimming, de novo assembly and mapping

Illumina paired-end sequencing generated 525,605,992 raw reads (244,543,276 and 281,062,716 raw reads from ovary and testis samples, respectively), corresponding to 26.30 GB of sequence data (12.10 GB and 14.20 GB from ovary and testis, respectively). Raw reads are stored into the NCBI Sequence Read Archive database under accession numbers SRR8631955-SRR8631960. After the trimming and filtering steps a total of 196,658,624 cleaned reads were recovered from female and 245,684,318 from male samples, for a total of 442,342,942 reads survived the processing. De novo Trinity assembly from both ovary and testis reads together produced 328,495 assembled transcripts. BUSCO revealed a 97.6% of transcriptome completeness, indicating a high quality de novo assembly. As the proportion of duplicates was high (64%), the EvidentialGene tr2aacds pipeline was used to reduce the transcriptome redundancy. The non-redundant transcriptome consisted in 79,796 transcripts with also a high level of completeness (97%), and only a resulting 4.6% of duplicates. De novo assembly statistics are summarised in Table 1. All female and male reads were mapped back together on the non-redundant transcriptome and only transcripts with an expression value of TPM ≥ 1 were included in the final set of transcripts. After removing ribosomal- and mitochondrial-related transcripts, the final ovary and testis non-redundant transcriptome consisted in 39,186 transcripts.

**Table 1:** Summary statistics of *Palaemon serratus* gonads transcriptome sequencing and assembly

	Ovary samples	Testis samples	Redundant transcriptome	Non-redundant transcriptome
Number of raw reads	244,543,276	281,062,716	–	–
Raw reads length	101	101	–	–
Number of reads after trimming	196,658,624	245,684,318	–	–
Reads length after trimming	40–86	40–86	–	–
Number of Trinity genes	–	–	165,152	69,481
Number of Trinity transcripts	–	–	328,495	79,796
Total size of transcripts (bp)	–	–	268,430,756	77,857,728
Mean transcript size (bp)	–	–	817.15	975.71
N50 transcript length (bp)	–	–	1744	1996
Assembly completeness	–	–	97.60%	97.0%
Assembly duplication	–	–	64.0%	4.6%

### Functional annotation

A total of 11,586 assembled transcripts (29.57%) were annotated at least against one of the used databases (Additional file 1: Table S1). Therefore, the 70.43% of the ovary and testis transcriptome remained unannotated. Number of transcripts matching each annotation category is listed in Table 2. BLASTx searches against the UniProtKB/Swiss-Prot database identified 17,285 transcripts from 437 species. As UniProtKB/Swiss-Prot is a reviewed database, the BLASTx top hit species were model species, e.g. *Homo sapiens* (2547 sequence hits), *Mus musculus* (1749 hits), *Drosophila melanogaster* (1619 hits), *Rattus norvergicus* (581 hits) or *Bos taurus* (540 hits). TransDecoder found 18,312 putative ORFs (Open Reading Frames) in the assembled transcripts and about 59% of them were full-length. A total of 15,961 out of these protein-coding candidate transcripts returned a BLASTp hit when they were searched against the known proteins of the UniProtKB/Swiss-Prot database. Searches against the PFAM protein domain database retrieved 182,498 hits on the putative peptides. Of these ORFs, 1492 were predicted to contain a secretion domain and 2790 were predicted to contain at least one transmembrane helix domain. The assembled transcripts were also annotated with Gene Ontology (GO) terms according to the three major GO categories: cellular component, molecular function, and biological process. A total of 13,960 GO terms were assigned and 6846 transcripts (17.47% of the transcriptome) were associated with at least one term. Accurately, 1809 transcripts were assigned to a cellular component category, 5952 to a molecular function category, and 3155 to a biological process category.

**Table 2:** Overview of functional annotation results

	Number of transcripts
UniProtKB/Swiss-Prot BLASTx hits	17,285
ORF-containing transcripts:	18,312
5′ partial	3340
internal partial	2468
complete	10,843
3′ partial	1661
UniProtKB/Swiss-Prot BLASTp hits	15,961
PFAM hits	182,498
Signal peptide domains	1492
TmHMM domains	2790
GO assigment:	6846
Cellular Component	1809
Molecular Function	5952
Biological Process	3155

### Differential expression and enrichment analyses

Clean reads from each sample were mapped back on the non-redundant ovary and testis transcriptome. The percentage of mapped reads oscillated from 65.66 to 73.34% among samples. At the same time, larvae and muscle reads downloaded from the SRA (SRR4341161–2 and SRR4341163–4, respectively) were cleaned and also mapped separately on the ovary and testis transcriptome in order to identify genes with a gonad-biased expression. The percentage of mapping ranged from 27.83% mapped reads for larvae to 32.68% mapped reads for muscle. Gene expression of the assembled transcripts in the larvae and muscle samples was calculated and pairwise differential expression analyses (DEAs) were performed between gonad and non-gonad samples (ovaries vs. larvae, ovaries vs muscle, testes vs larvae and testes vs muscle). Genes with a False Discovery Rate (FDR) *p*-value ≤0.01 and a fold-change > 2 were consider significatively up-regulated in the gonad tissues respecting to the non-gonad tissues. A total of 1961 and 774 genes were identified as up-regulated in ovaries with regard to larvae and to muscle, respectively. As for testes, 1338 and 1118 genes were detected as up-regulated with regard to larvae and to muscle, respectively. By removing duplicated up-regulated genes shared by both female and male gonads, 3646 genes were considered as up-regulated genes in both gonads and so they were tagged with a ‘G’ (gonad up-regulated) in the transcriptome annotation table (Additional file 1: Table S1).

A DEA was carried out between ovary and testis libraries to identify DEGs between sexes and therefore, putative sex-related genes. Transcripts with a FDR *p*-value ≤0.01 and an absolute value of fold-change > 2 were considered to be significative DEGs. Overall, 11,087 transcripts were identified as DEGs (Additional file 2: Figure S1). Among these DEGs, the 39.09% out of them had an annotation from at least one of the used databases, and 6207 genes were up-regulated in ovaries meanwhile 4880 genes were up-regulated in testes (Additional file 1: Tables S2 and Additional file 1: Table S3). A gene was considered ovary- or testis-specifically expressed when its TPM value were less than 1 in the three testis or ovary samples, respectively. The 78.22% of the genes previously identified as gonad up-regulated genes (G-genes) matched with DEGs between ovaries and testes. Fine scale comparative results from the different DEAs are shown in Table 3.

**Table 3:** Results of Differential Expression Analysis between ovary and testis simples

	Number of transcripts
Ovary and testis transcriptome	39,186
DEGs between ovary and testis:	11,087
Up-regulated genes in ovary	6207
Ovary-specific genes	2997
Up-regulated genes in testis	4880
Testis-specific genes	2853
Gonad up-regulated genes (G)	3646
G-genes and DEGs between ovary and testis:	2852
Up-regulated genes in ovary	1708
Ovary-specific genes	712
Up-regulated genes in testis	1144
Testis-specific genes	400

**Fig. 1:**
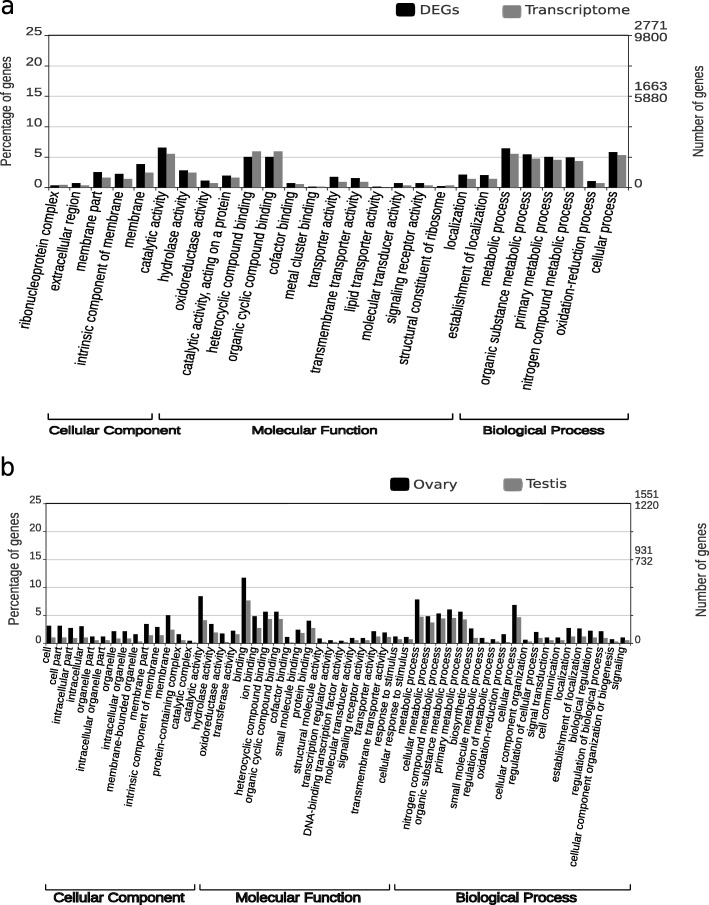
**a** Comparative distribution of GO terms between DEGs and the entire ovary and testis transcriptome. **b** Comparative distribution of GO terms between up-regulated genes in ovary and up-regulated genes in testis

Comparative GO classification distribution of the annotated genes showed no large differences between DEGs and the entire gonadal transcriptome (Fig. 1a). Statistically, GO enrichment analyses were performed and a *p*-value ≤0.01 was considered as threshold to identify putative functional differences between DEGs and the ovary and testis transcriptome. Based on these GO analyses, 3 GO terms were found to be significantly enriched in DEGs in the cellular component category, ‘extracellular region’, ‘integral component of membrane’ and ‘membrane’. There were 18 significantly enriched GO terms in DEGs at the molecular function category, being the most enriched terms ‘ionotropic glutamate receptor activity’, ‘chitin binding’ and ‘G protein-coupled receptor activity’. As for the biological process level, 8 GO terms showed significative enrichment in DEGs, corresponding the most enriched assignations to ‘chitin metabolic process’, ‘transmembrane transport’ and ‘DNA integration’. Complete results from the GO enrichment analyses are included as Additional file 1: Table S4. No remarkable differences between ovary and testis up-regulated genes were detected when their GO term distributions were plotted (Fig. 1b).

### Candidate DEGs to be involved in sexual development

We aimed to reveal genes involved in sex determination, sex differentiation and gonadal development pathways. To achieve this purpose, it is crucial to explore the DEGs between sexes. Thus, up-regulated expressed genes in ovaries and in testes were mined according to the transcriptome annotation and the published literature in search of putative sex-related genes. When different transcripts matched the same gene annotation their BLAST hits were manually cured, and then the full-length transcript was chosen or, in the case that all of them were complete ORFs, the transcript with the highest expression was selected.

### Up-regulated genes in ovary

The most expressed up-regulated genes in ovary libraries generally retrieved no annotations that allowed us to identify them. These top expressed genes mostly matched with genes tagged as ‘gonad up-regulated’ when they were compared with non-gonad tissues (Additional file 1: Table S2). The most highly expressed up-regulated genes that could be annotated were cytochrome c oxidase subunit 3 (Cox3), cellular retinoic acid-binding protein 2 (Crabp2), ferritin, death-associated inhibitor of apoptosis 1 (Diap1), ankyrin-like, annexin and NPC intracellular cholesterol transporter 2 homolog a (Npc2). However, our focus was to examine candidate genes to be involved in female sexual development within the 6207 up-regulated genes in ovary. A total of 15 sex-related DEGs were found to be up-regulated in ovary samples, being 10 out of them also gonad up-regulated genes and 3 considered ovary-specific genes (Table 4). Genes associated with vitellogenesis and ovary development were the DEGs that showed the higher expression levels: vitellogenin (Vg), vitellogenin receptor (VgR), cathepsin D, chorion peroxidase (Pxt) and profilin. Among up-regulated DEGs, they were also present prostaglandins metabolism-related genes, prostaglandin D synthase (Hpgds) and prostaglandin E synthase 2 (Pges2), along with farnesoic acid-O-methyl transferase (FAOMeT), heat shock cognate 70 (Hsc70), mothers against decapentaplegic 4 (Smad4), gonadotropin-releasing hormone receptor (Gnrhr), and progestin membrane receptor component 1 (Pgmrc1). Feminization-1b (Fem-1b), disrupted meiotic cDNA (Dmc1) and transcription factor Sox8 were detected as up-regulated genes in the ovary even though they were traditionally associated to male sexual development in previous studies (see Discussion).

**Table 4:** Differentially expressed genes candidates to be involved in female sexual development

Gene	Gene annotation	Sequence ID	Length (bp)	ORF	Fold change (ovary/testis)	FDR *p*-value	Mean TPM ovaries	Mean TPM testes	Gonad up-regulated
Vg	Vitellogenin	TRINITY_DN69557_c0_g1_i2	7827	complete	8.53	9.16E-6	635.87	106.46	yes
Vgr	Vitellogenin receptor	TRINITY_DN75859_c0_g1_i3	5765	complete	61.24	0.00	754.94	16.39	yes
Hpgds	Hematopoietic prostaglandin D synthase	TRINITY_DN66544_c0_g1_i1	2050	complete	4.98	6.96E-3	107.45	26.71	yes
Cathepsin D	Cathepsin D	TRINITY_DN74459_c2_g2_i1	2138	complete	16.92	9.31E-8	2637.5	224.02	yes
Pxt	Chorion peroxidase-like	TRINITY_DN71711_c0_g1_i1	2419	complete	4872.33	0.00	2495.51	0.67	yes
Fem-1b	Protein fem-1 homolog B	TRINITY_DN63971_c0_g1_i2	3646	complete	2.65	6.87E-3	37.10	19.73	no
Profilin	Profilin	TRINITY_DN61540_c0_g3_i2	904	complete	38.02	0.00	1768.14	68.09	yes
FAOMeT	Farnesoic acid-O-methyl transferase	TRINITY_DN64401_c1_g5_i1	1726	complete	7.84	3.52E-6	841.08	154.89	yes
Hsc70	Heat shock cognate 70	TRINITY_DN71006_c1_g1_i1	2040	3′ partial	3.64	4.79E-03	674.77	259.44	no
Dmc1	Disrupted meiotic cDNA	TRINITY_DN62322_c4_g1_i1	1526	complete	5.30	9.59E-5	222.61	60.22	yes
Sox8	Transcription factor SOX-8	TRINITY_DN70088_c0_g1_i1	4022	complete	7.26	1.99E-3	3.57	0.58	no
Smad4	Mothers against decapentaplegic 4	TRINITY_DN62236_c4_g2_i4	3768	complete	24.67	0.00	97.11	4.91	yes
Gnrhr	Gonadotropin-releasing hormone receptor	TRINITY_DN64884_c0_g1_i3	2199	complete	2493.02	0.00	62.82	0.03	yes
Pges2	Prostaglandin E synthase 2	TRINITY_DN55480_c0_g1_i1	1590	complete	4.00	2.74E-4	51.2	18.82	no
Pgmrc1	Progestin membrane receptor component 1	TRINITY_DN74305_c2_g1_i3	1763	complete	3.17	9.65E-3	17.48	7.48	no

### Up-regulated genes in testis

Up-regulated genes in testis that showed the highest expression values also remained unannotated but they were identified as gonad up-regulated genes (Additional file 1: Table S3). The most expressed up-regulated annotated genes in testis were Kazal-like protease inhibitor MCPI, tenascin-X (TnxB), histone H1-delta (H1D), myosin light chain kinase (Mylk), histone H2A (H2A), RNA-directed RNA polymerase L and protein innexin-like. Regarding to DEGs associated with male sexual development, within the 4880 up-regulated genes in testis we identified 6 sex-related genes, being 2 out of them gonad up-regulated genes and 5 considered as testis-specific genes (Table 5). Doublesex- and mab-3-related transcription factor 1 (Dmrt1) was the sex-related up-regulated gene with the highest expression. As Dmrt1, three transcription factors belonging to the SOX (Sry-related HMG box) family showed testis-specific expression, Sox5, Sox14 and Sox15. There were also up-regulated genes in testis that did not agree with the traditional literature about male sexual development, namely vitelline membrane outer layer protein 1 (Vmo1) and heat shock protein 90 (Hsp90) (see Discussion).

**Table 5:** Differentially expressed genes candidates to be involved in male sexual development

Gene	Gene annotation	Sequence ID	Length (bp)	ORF	Fold change (ovary/testis)	FDR *p*-value	Mean TPM testes	Mean TPM ovaries	Gonad up-regulated
Dmrt1	Doublesex- and mab-3-related transcription factor 1	TRINITY_DN68477_c1_g1_i10	2920	complete	−17.59	7.37E-9	19.54	0.82	yes
Vmo1	Vitelline membrane outer layer protein 1	TRINITY_DN62635_c0_g1_i2	1495	complete	−9.18	4.01E-6	16.91	1.26	yes
Sox15	Putative transcription factor SOX-15	TRINITY_DN73558_c1_g2_i1	1699	3′ partial	−11.60	1.90E-4	5.37	0.38	no
Sox5	Transcription factor SOX-5	TRINITY_DN67052_c2_g1_i1	6054	5′ partial	−5.31	1.85E-4	5.13	0.69	no
Hsp90	Heat shock protein 90	TRINITY_DN69393_c0_g1_i1	2573	complete	−5.00	7.64E-3	3.66	0.52	no
Sox14	Putative transcription factor SOX-14	TRINITY_DN52668_c0_g1_i1	1704	5′ partial	−14.07	1.66E-3	3.00	0.17	no

### Not differentially expressed genes

Several genes that have been largely dealed as sex-related genes in crustaceans were investigated even though they were not DEGs in the present study (Table 6). No differential expression of these genes might respond to they are not involved in sexual development or because the differential expression took place in gonads before dissection. In detail, these genes comprised genes that were reported to act either triggering female or male determination or sex differentiation (e.g. sex-lethal, feminization-1a and -1c or forkhead box L2) or taking part in specific processes later on ovary (e.g. estrogen-related receptor or follistatin) or testis (e.g. kinesin-like protein KIFC1) development and maturation. Due to the expression of some sex-related genes was TPM < 1, they were not included in the final transcriptome. These genes with an extremely low expression were doublesex and mab-3-related transcription factor 11E (Dmrt11E), insulin-like androgenic factor (Iag) and Wnt4 transcription factor. Other interesting genes in reproduction with a documented preferential expression in non-gonad tissues were detected in our gonadal transcriptome, two members of the crustacean hyperglycemic hormone superfamily (Chh and Mih) and the ecdysone receptor (EcR) gene.

**Table 6:** Inventory of sex-related genes not differentially expressed between sexes in this study

Gene	Gene annotation	Sequence ID	Length (bp)	ORF	FDR *p*-value	Gonad up-regulated
β-catenin	Beta-catenin	TRINITY_DN68450_c3_g1_i1	4487	complete	0.28	no
Err	Estrogen-related receptor	TRINITY_DN62849_c1_g1_i1	3564	complete	0.02	no
Fem-1a	Feminization factor 1a	TRINITY_DN72410_c0_g1_i3	2424	complete	0.27	no
Fem-1c	Feminization factor 1c	TRINITY_DN67155_c0_g1_i1	4405	complete	0.05	no
Foxl2	Forkhead box L2	TRINITY_DN67281_c5_g1_i1	3329	complete	0.95	yes
Fst	Follistatin	TRINITY_DN65522_c1_g2_i4	3175	complete	0.22	no
Kifc1	Kinesin-like protein KIFC1	TRINITY_DN70471_c2_g1_i10	3013	complete	0.25	no
Msl3	Male-specific lethal 3	TRINITY_DN62887_c0_g2_i3	3270	complete	0.20	no
Ptgr1	Prostaglandin reductase 1	TRINITY_DN59739_c0_g1_i3	1538	complete	0.99	no
Sxl	Sex-lethal	TRINITY_DN60874_c2_g1_i16	1340	complete	0.46	no
Tra-2	Transformer 2	TRINITY_DN66599_c7_g4_i2	1920	3′ partial	0.31	no
Vasa	Vasa	TRINITY_DN61866_c4_g1_i1	2619	complete	0.73	yes
Dmrt11E*	Doublesex- and mab-3-related transcription factor 11E	TRINITY_DN65796_c0_g1_i1	2931	complete		
Iag*	Insulin-like androgenic gland factor	TRINITY_DN53120_c0_g1_i3	433	internal		
Wnt4*	Wingless-type MMTV integration site family, member 4	TRINITY_DN24615_c0_g1_i1	425	internal		
Chh	Crustacean hyperglycemic hormone	TRINITY_DN70124_c2_g1_i2	844	complete	0.93	no
Mih*	Molt inhibiting hormone	TRINITY_DN72393_c0_g2_i1	755	complete		
EcR	Ecdysone receptor	TRINITY_DN75882_c1_g1_i6	5148	complete	0.96	no

Asterisk indicates genes with a TPM value < 1

Sequences of the sex-related genes listed in Table 4, Table 5 and Table 6 can be easily access in Additional file 3: File S1.

Finally, there were sex-related genes whose expression was not detected neither in the non-redundant transcriptome nor in the redundant one. This fact could be explained because the expression of these genes occurs in other tissues, or because they were expressed in gonads but not at the stage when animals were dissected or because they are not actually sex-regulators in *P. serratus*. Relevant non-expressed sex-related genes were doublesex (Dsx), fruitless (Fru), sex-determining region Y (Sry), transcription factors Sox9 and Sox10, cytochrome P450 aromatase, R-spondin-1 (Rspo1), steroidogenic factor 1 (Sf-1) and fibroblast growth factor 9 (Fgf9).

## Discussion

*Palaemon serratus* is a relevant commercial species in some countries such as UK, Ireland, France, Spain and Portugal [21]. The lack of genomic information about *P. serratus* hinders the application of potential aquaculture techniques, especially those focused on reproductive traits related to sex dimorphism. Therefore, in the present work we attempted to unravel sex-related genes featured in sex determination, sex differentiation and/or gonadal development using a RNA-Seq approach. This is the first transcriptome analysis of the gonads of a *Palaemon* species and the first work that provides data about sex-related genes in *P. serratus*. Within Palaemonidae, sex-related genes have been only studied in two *Macrobrachium* species [8, 9, 22]. Here, a reference gonadal transcriptome was obtained using ovary and testis reads. Statistics indicated a high quality de novo assembly but only the 29.57% of the transcriptome could be annotated due to the scarcity of crustacean genomic sequences. Precisely, the most expressed up-regulated genes both in ovary and in testis were not annotated. As the non-annotated transcripts correspond to unknown novel transcripts or to unreviewed transcripts, these unannotated differentially expressed sequences deserve attention in future functional analyses about sex-related genes in this shrimp. Additionally, the complete transcriptome annotation is provided, largely increasing the sequence resources available for this species.

Sexual development includes several processes orchestrated by a variety of regulators. Overall, sex determination and sex differentiation are intricated processes not always clearly distinguishable because their signalling cascades can be integrated [11]. Sex determination mechanisms are widely divergent in animals, even between closely related species. This variability is due to the rapid evolution that sex-biased genes experience [11, 23] and is reflected to crustaceans, where is not uncommon for findings in sex-related genes to differ among species. This can be linked to there is no conserved sex determination pathway in decapods and it likely evolved independently several times, making difficult to trace master sex-regulators [24]. Back to *P. serratus*, heteromorphic sex chromosomes are absent [20] and none sex determination system has been documented. It was suggested that there is a sex chromosome dosage compensation mechanism involving Msl3 gene in the tissues of the palaemonid *Macrobrachium nipponense* [25] as in *Drosophila*. Unfortunately, Msl3 was not a DEG between females and males in *P. serratus*, giving no hint to whether a heterogametic sex exists in this shrimp or not and what is it. Hence, we focused our efforts to study genes described as ‘sex-related’, mainly in crustaceans.

Orthologs of sex determination genes in the model arthropod *Drosophila* were found in our transcriptome database. Sxl and Tra-2 orthologs were detected without sex differential expression meanwhile Tra, Dsx and Fru were absent. In *Drosophila* sex is ruled by the genetic pathway Sxl-Tra/Tra-2-Dsx/Fru, being Sxl the master sex determinant gene [12, 26]. It has been proposed that crustaceans may adopt the *Drosophila* sex determination pathway given the findings reported in some species as *Penaeus monodon* [27], *Macrobrachium nipponense* [28], *Penaeus chinensis* [29], *Penaeus vannamei* [30] and *Eriocheir sinensis* [31]. However, our data are in line with those that stand that these genes do not act in decapods as they do in insects (see review in [7]), as suggested for the lobster *Sagmariasus verreauxi* [32] or for the crab *Scylla paramamosain* [33].

In the nematode *Caenorhabditis elegans*, Fem-1 is a component of the sex determination signalling pathway that promotes the male phenotype [34, 35]. There are studies in decapods that pointed out the putative role of Fem-1 genes in male sex determination [18, 36]. Orthologs of the three members of the Fem-1 family were found in *P. serratus* but none of them was up-regulated in the testis. Fem-1a and Fem-1b were not DEGs between sexes whilst Fem-1b was found to be slightly up-regulated in the ovary. Particularly to Fem-1b, [37] detected a higher expression of this gene in the testes than in the ovaries of the prawn *Macrobrachium nipponense*, which is the opposite of what we found in *P. serratus*. Also in *M. nipponense,* [38] reported a ovary-specific Fem-1 gene that could be involved in sex determination or differentiation and in ovarian maturation in this species. Fem-1b ortholog was up-regulated in the ovary of *P. serratus* but it was not an ovary-specific gene given that it also exhibits a considerable expression in testis. We conclude that similarly to in *Scylla paramamosain* [17] and in *Penaeus vannamei* [39], our results seem to indicate that whether Fem-1 genes are involved in the sexual development in *P. serratus* has yet to be established given that they are expressed in both gonads.

The male-determining gene in most mammals is the Y chromosome Sry gen [40, 41]. SRY along with SF-1 induces testicular development through the activation of the transcription factor SOX9 [42]. SOX9 up-regulates via FGF9 the expression of the Dmrt1 gene, which is the major male sex differentiation gene, promoting testis development and maintenance [43]. Expression of Sry, Sf-1, Sox9 and Fgf9 was not detected in the gonads of *P. serratus.* Some members of the Sox family were DEGs, Sox8 was up-regulated in ovaries and Sox5, Sox14 and Sox15 were specifically expressed in testes. The up-regulation of Sox8 in females was unexpected as this gene has been related with testis development [12, 32]. Both Sox5 and Sox14 were previously identified as genes involved in male sex differentiation with expression in testis tissues [15, 44–46] but Sox15 has never been defined as a testis gene before. Nevertheless, the most relevant finding in *P. serratus* gonadal transcriptome is the testis-specific expression of the Dmrt1 gene.

In some vertebrate species Dmrt1 has been qualified as a sex-determining gene [47, 48]. Moreover, Dmrt1 paralogs are the master sex determinant genes in medaka fish [49], frogs [50] and, recently in *Sagmariasus verreauxi* [11] as the first invertebrate species in which Dmrt1 determinates the sex. DMRTs are transcription factors characterized by the presence of a DM domain DNA binding motif. The relationship between DM domain genes and sex has been deeply investigated and as it is thought that their ancestral function was likely to determine gonadal sex and they subsequently expanded to control sexual dimorphism in other tissues [43]. Dmrt is the only gene family with a conserved function in sex determination across *Animalia* [11] and orthologs have been identified with a testis-restricted expression in the transcriptome of a few decapod species as the crabs *Eriocheir sinensis* [16] and *Scylla paramamosain* [17]. Keeping this in mind, the testis-specific Dmrt1 ortholog found in *P. serratus* should be considered the best candidate gene to be involved in the sex determination of this species. Future efforts should be directed to functionally characterize the Dmrt1 gene and to pursue upstream regulators and downstream targets. Another Dmrt gene was found in the transcriptome with an extremely low expression (TPM < 1), the Dmrt11E gene that has been previously detected in some decapods. This gene exhibited a testis-biased expression in *Macrobrachium rosenbergii* [51] and an androgenic gland-biased expression in *Sagmariasus verreauxi* [32], and in both cases it was suggested that Dmrt11E is a male differentiation regulator via IAG. As the Dmrt11E expression in *P. serratus* gonads is very low, another tissue should be the primary site of expression instead of testis, likely the AG. Owing to its proved relationship with the IAG in other species, expression of Dmrt11E should be studied in different organs of *P. serratus*.

The IAG is the key regulator of male sex differentiation in the members of Malacostraca and its expression takes place exclusively in the androgenic gland (AG) of males. The Iag gene was characterized in several crustaceans, e.g. in the prawn *Penaeus monodon* [52], in the shrimps *Penaeus vannamei* [53], *Macrobrachium lar*, *Palaemon paucides* and *P. pacificus* [54], or in the spiny lobsters *Sagmariasus verreauxi* and *Jasus edwardsii* [32]. The expression level of the Iag gene in our gonadal transcriptome was very low (TPM = 0.84), likely because in *Palaemon* species the AG is located along the sperm ducts and not in the testis [54], and they should not be dissected along with the testis. A better knowledge about Iag is crucial because its genetic manipulation-based biotechnology has the potential to dramatically transform the entire aquaculture industry [55]. Monosex culture has the potential to enhance the production because energy for reproduction is allocated to growing, so the individuals reach higher sizes [54]. As female shrimps grow larger and faster, all-female population cultures are preferred for *P. serratus.* It has been proved in different decapod species that AG removal feminizes males [13], but this surgical procedure frequently entails mortality, so get monosex cultures by genetic manipulation is highly attractive. All-male populations were achieved for *Macrobrachium rosenbergii* silencing Iag using RNA interference [14]. To obtain all-female populations in *P. serratus* we suggest exploring the manipulation of the Iag gene to induce female sex-reversal. We provide the first Iag sequence for *P. serratus* and, even though is a partial sequence, it has the potential to pave the way to further biotechnological approaches that enable the production of female monosex cultures. These aquaculture strategies may enhance *P. serratus* production and at the same time prevent the genetic deterioration of the wild stocks caused by overfishing.

Genes referred in literature as ‘testis development’ genes were also investigated. KIFC1 is a C-terminal kinesin motor protein that participates in acrosome biogenesis and nuclear reshaping during spermiogenesis in the palaemonids *Macrobrachium nipponense* [56] and *Palaemon modestus* [57] among other crustacean species. Kifc1 gene showed a high expression in the testis of *M. nipponense* and *P. modestus* but in both species this gene was also being expressed in other tissues, likely taking part in vesicle transportation processes [56], so is not strange that Kifc1 was not a DEG between ovary and testis in *P. serratus*. Temporal and spatial expression of Kifc1 during spermiogenesis in *P. serratus* could be address since its protein is vital in the formation of the acroframosome, an exclusive structure of caridean shrimp spermatids. Concerning to DMC1, it plays a major role in meiotic recombination and has been associated to spermatogenesis in crustaceans [27]. Unlike in the crawfish *Procambarus clarkii* [18] and the crab *Portunus trituberculatus* [58], Dmc1 was not up-regulated in the testis but in the ovary of *Palaemon serratus*. It is important to highlight that Dmc1 was a gonad up-regulated gene, most likely because it is expressed in meiotic germ cells [59], but its role in the spermatogenesis of *P. serratus* should not be directly attributed without further testing. Another gene related with germ cell development is vasa, an ATP-dependent RNA helicase. Since vasa plays a role in both oogenesis and spermatogenesis its expression was exclusively detected in the gonads of the shrimp *Penaeus vannamei* [60] or of the crab *Scylla paramamosain* [61]. In this sense it was not surprising that vasa was a gonad up-regulated gene respect to non-gonad tissues but not a DEG between the ovary and the testis of *P. serratus.*

Regarding to female sex determination, Foxl2 gene encodes a conserved forkhead transcription factor preferentially expressed in the ovary of vertebrates, controlling the ovarian differentiation and maintenance by repression of testis-specific genes [62, 63]. If Dmrt1 is present, Foxl2 expression is repressed, but in the absence of Dmrt1, Foxl2 inhibits the male developmental pathway and promotes the female. The expression of Foxl2 showed a changing pattern among crustacean species, i.e. up-regulated in ovary [18], not DEG between sexes [31] or even up-regulated in testis [64]. Foxl2 was not a DEG between ovary and testis in *P. serratus,* which supports that the role of Foxl2 in sex determination in invertebrates remains unclear [65, 66]. Dmrt1 also acts repressing the RSPO1-WNT4-β-catenin signalling pathway, another female sex determination cascade that promotes ovary development in vertebrates independently and complementary to the Foxl2-leading pathway [67–69]. Given that Rspo1 was not found in the transcriptome of *P. serratus* and Wnt4 and β-catenin were detected as not DEGs, the existence and function of this pathway is unknown in this shrimp, as it was already advanced for other decapods with orthologs found [31, 33]. Thereby, vertebrate pathways leadered by Foxl2 and Rspo1 do not seem to determine female development in *P. serratus* in the light of our data. However, further experiments should confirm these lacks of function in sexual development.

Vitellogenesis, the production and accumulation of yolk, is crucial to oogenesis and ovarian maturation. In oviparous vertebrates vitellogenin synthesis is enhanced by 17β-estradiol E2, with the estrogen receptor (ER) and the HSP90 acting as mediators [70–73]. The elements of the E2-ER-HSP90 pathway were found in the transcriptome of decapod species [7, 64, 74] but only Hsp90 showed a higher expression in ovaries than in testes in the crab *Scylla paramamosain* [17]. An estrogen-related receptor gene (Err) was found in *P. serratus* without differential expression and Hsp90 was up-regulated in the testicular tissue, so our results agree that more studies are necessary to clarify if the E2-ER/ERR-HSP90 pathway exists in crustaceans and whether vitellogenesis it is regulated by estrogen-like hormones as it is in vertebrates. Another regulatory pathway that stimulates ovarian development and vitellogenesis in some decapods involves methyl farnesoate (MF), a crustacean juvenile hormone analogue [75, 76]. Farnesoic acid-O-methyl transferase (FAOMeT) encondes the enzyme that catalyzes formation of MF and it was up-regulated in the ovaries of *P. serratus* respect to testes and non-gonad tissues, indicating the putative role of this hormone in the ovarian maturation of the species. Vitellogenin (Vg) and vitellogenin receptor (VgR), the main vitellogenesis genes, were up-regulated in the ovary of *P. serratus* as in multiple decapod species (e.g. [24] or [58]). The expression of VgR was higher than the expression of Vg, likely because the hepatopancreas is considered the primary site of VG production instead of the ovary while the VGR allow VG uptake from the hemolymph by oocytes. Vitelline membrane outer layer protein 1-like gene (Vmo1) was strangely up-regulated in males, a finding also reported for the crab *Eriocheir sinensis* [31].

Other ‘ovary development’ genes were also explored in the gonadal transcriptome of *P. serratus.* Since prostaglandins (PGs) have been described as factors that promote ovary development in crustaceans [77], PG genes were studied in *P. serratus.* HPGDS and PGES2 showed an ovary-biased expression, so they might have an implication in female gonad development [78, 79]. Although PTGR1 is involved in the ovary development in the shrimp *Penaeus monodon* [80], it was not preferentially expressed in the ovaries of *Palaemon serratus*. Cathepsins have been also related with ovarian development in some crustaceans [22, 81, 82]. Cathepsin D is a needed protein for the formation of the yolk in vertebrates [83] and its gene was the only cathepsin gene up-regulated in the ovary of *P. serratus*. Other up-regulated genes in the ovary of *P. serratus* that are required for ovary maturation in other crustaceans were chorion peroxidase [31], profilin [84], Smad4 [85], Gnrhr [86] and Pgmrc1 [87]. Expression of Hsc70 was also up-regulated in the ovary of *P. serratus*, suggesting a putative role in reproductive events as in *Macrobrachium rosengergii,* where Hsc70 was enriched in the ovary [88]. Fst is known to be involved in folliculogenesis and ovary development in vertebrates [89], but it was not a DEG between the gonads of *P. serratus,* similarly to *Eriocheir sinensis* [31]*.* The expression of Fst should be examined throughout the stages of the ovarian development to evaluate whether it participates in it or not. Cytochrome P450 aromatase (Cyp19a) is also essential in the female gonadal development in vertebrates, converting androgens in estrogens. Several genes belonging to cytochrome P450 superfamily were detected in the transcriptome of *P. serratus*, but none of them was aromatase, an absence also found in the palaemonid *Macrobrachium rosenbergii* [90].

Lastly, two genes encoding two members of the CHH-superfamily were detected in the gonadal transcriptome with a very low and not differential expression between sexes: the crustacean hyperglycemic hormone (Chh) and the molt inhibiting hormone (Mih). CHH neuropeptides are multifunctional hormones with roles in reproduction, regulating AG proliferation or MF production among other activities (see review in [77]). The eyestalk is the preferential site of production of these neurohormones, but it was repeatedly demonstrated that these genes are also expressed in multiple tissues [91–93], including gonads in some species as in *P. serratus*. Likewise, as recent studies have demonstrated that ecdysteroids regulate vitellogenesis, ovarian maturation and spermatogenesis in decapods (see review in [78]), it is also interesting to highlight the expression of the ecdysone receptor (EcR) gene in both female and male *P. serratus* gonads.

## Conclusions

This study encompasses the first large-scale RNA-Seq and comprehensive transcriptome analysis of *Palaemon serratus* gonads. More than 442 million of clean reads were obtained, 39,186 transcripts were assembled and annotated and 11,087 out of them were found to be DEGs between ovary and testis. Sex-related genes were identified and their expression between sexes was studied. A wide inventory of sex-related genes is provided and thoroughly discussed in the framework of previous findings in other crustacean species. This is the first time that sex-related genes have been addressed in a *Palaemon* species, so this transcriptomic analysis will facilitate further experimental research that aimed to delve into the sex determination, sex differentiation and gonadal development mechanisms of *P. serratus* and close species. The candidate genes to be involved in sexual development might also shed light about the evolution of sex-regulators in crustaceans. Furthermore, we report some particularly interesting genes towards investigating future aquaculture applications for *P. serratus*.

## Methods

Specimens of *P. serratus* used in this study were collected inshore from the Ártabro Gulf (43°22′00″N, 8°28′00′W) in the northwest of Spain using a fish trap. Animals were carried alive to the Aquarium Finisterrae dependencies (A Coruña, Spain) where they were kept at 18 °C in an aerated aquarium while they were sorted into sexes. According to [3], sex was determined by the presence (in males) or absence (in females) of the masculine appendix on the endopodite of the second pleopod. Shrimps (3–4.5 g body weight) were anesthetized on ice for 5 min prior to be sacrificed by dissection, and then gonads from three adult females and three adult males were quickly removed and directly immersed in liquid nitrogen. The development stage of the gonads was fully mature in all individuals. RNA isolation and library construction were carried out at AllGenetics & Biology SL (A Coruña, Spain). Total RNA was extracted from the six samples by grinding them with a mortar and pestle under liquid nitrogen. The resulting powder was used for the extraction using NZYTech’s Total RNA isolation kit (NZYTech). Pure RNA was eluted in a final volume of 30 μL and then quantified and quality-checked in an Agilent 2100 Bioanalyzer (Agilent) using the Agilent RNA 6000 Kit (Agilent). Illumina’s TruSeq Stranded mRNA Library Prep Kit (Illumina) was used to prepare six cDNA libraries following the manufacturer’s instructions. Thus, one library per sample was prepared, i.e. three libraries from ovary tissues and three libraries from testis tissues were prepared, or in other words three biological replicates per sex. The fragment size distribution and concentration of the libraries were checked in the Agilent 2100 Bioanalyzer using the Agilent DNA1000 Kit (Agilent). Libraries were quantified using the Qubit dsDNA BR Assay Kit (ThermoFisher Scientific). All libraries were pooled in equimolar amounts, according to the quantification data. The pool was sequenced in two different lanes of a HiSeq 4000 PE100 platform (Illumina).

Raw reads quality control was performed using FastQC v0.11.5 [94]. Trimmomatic v0.35 [95] was used for raw reads trimming. Primer/adaptor sequences were removed and the first 15 bp of the reads were cut. Trimmed reads shorter than 40 bp were discarded. Both female and male gonads trimmed reads were assembled together to obtain a single transcriptome that included ovary and testis transcripts. Trinity software v2.4.0 [96] was used for de novo assembly of these high-quality short reads using default parameters settings (Kmer = 25). Assembled transcriptome completeness was assessed with BUSCO v3.0.2 [97] using the Arthropoda database as reference. EvidentialGene tr2aacds pipeline [98] was used to reduce the transcriptome redundancy. Then BUSCO was run again to check the duplication level. Gene expression given as Transcript Per Million (TPM) was calculated by mapping back all ovary and testis trimmed reads together on the gonads assembled transcriptome (parameters: minimum allowed length fraction = 0.75, similarity fraction = 0.95 and maximum number of matching contigs = 4) using the RNA-Seq tool of the CLC Genomics Workbench v11.0 (Qiagen). Only transcripts with a TPM ≥ 1 were included in the definitive transcriptome. Ribosomal and mitochondrial contigs were identified by BLASTn [99] against the NCBI non-redundant (nr) database and they were also excluded from the final transcriptome. Functional annotation was carried out using the Trinotate v3.1.1 workflow (https://trinotate.github.io). In detail, sequence similarity of the assembled transcripts was evaluated using BLASTx [99] against the UniProtKB/Swiss-Prot database (E-value cut-off of 1e-5). TransDecoder v5.3.0 [100] was used to identify putative protein coding regions, including homology options as retention criteria for the candidates ORFs. Predicted ORFs were identified by BLASTp [99] queries against the UniProtKB/Swiss-Prot database (E-value cut-off of 1e-5). Protein functional domains were identified using HMMER3 [101] against the PFAM domain database. Signal peptide and transmembrane domains prediction was performed with SignalP v4.1 [102] and TMHMM v2.0 [103], respectively. WEGO [104] was used to plot the Gene Ontology (GO) functional classification and distribution of the annotated transcripts.

Trimmed reads of each gonad sample were mapped back separately on this reduced high-quality set of ovary and testis transcripts (parameters: minimum allowed length fraction = 0.75, similarity fraction = 0.95 and maximum number of matching contigs = 4) using the RNA-Seq tool of the CLC Genomics Workbench v11.0 (Qiagen). Thus, expression of each transcript in each sample was calculated as Transcript Per Million (TPM). Subsequently, a DEA between ovary and testis samples was carried out in order to identify DEGs between ovaries and testes using the Differential Expression for RNA-Seq tool of the CLC Genomics Workbench v11.0 (Qiagen). GO enrichment analysis was conducted to reveal GO terms significantly enriched in DEGs using the CLC Genomics Workbench v11.0 (Qiagen). Transcriptomic SRA data (NCBI Sequence Read Archive) from other *P. serratus* tissues, larvae (SRR4341161–2) and muscle (SRR4341163–4), was used to identify differentially expressed genes (DEGs) between gonad tissues (ovary and testis) and non-gonad tissues (larvae and muscle). Firstly, SRA reads quality was checked with FastQC v0.11.5 [94] and Trimmomatic v0.35 [95] was used to trim the reads as follows: HEADCROP:15 TRAILING:25 MINLEN:40. SRA trimmed reads were mapped on the non-redundant and reduced gonads transcriptome to calculate the gene expression (TPM) of the assembled transcripts in the larvae and muscle samples. Pairwise differential expression analyses (DEAs) were performed between gonad and non-gonad samples (ovaries vs. larvae, ovaries vs muscle, testes vs larvae and testes vs muscle) using the CLC Genomics Workbench v11.0 (Qiagen). Up-regulated expressed genes in gonad tissues were tagged with a ‘G’ (gonad up-regulated) in the transcriptome annotation table.

## Supplementary information

**Additional file 1: Table S1.** Annotation table of the non-redundant *P. serratus* ovary and testis transcriptome. **Table S2.** Full list of up-regulated genes in ovary. **Table S3**. Full list of up-regulated genes in testis. **Table S4.** Results of GO enrichment analyses.

**Additional file 2: Figure S1.** Volcano plot of differentially expressed genes (DEGs) between ovary and testis samples. Not differentially expressed genes are shown with black dots meanwhile DEGs are depicted with red dots.

**Additional file 3: File S1.** Sequences of the discussed sex-related genes.

## Data Availability

The datasets generated and/or analyzed during the current study are available in the NCBI Sequence Read Archive (SRA) repository (accession numbers SRR8631955-SRR8631960) but restrictions apply to the availability of these data, and so they are not publicly available until article publication.

## Abbreviations

AG: Androgenic gland
Bp: Base pair
Chh: Crustacean hyperglycemic hormone
Cox3: Cytochrome c oxidase subunit 3
Crabp2: Cellular retinoic acid-binding protein
Cyp19a: Cytochrome P450 aromatase
DEA: Differential expression analysis
DEG: Differentially expressed gene
Diap1: Death-associated inhibitor of apoptosis 1
Dmc1: Disrupted meiotic cDNA
Dmrt: Doublesex- and mab-3-related transcription factor
Dsx: Doublesex
E2: 17β-estradiol E2
Ecr: Ecdysone receptor
Er: Estrogen receptor
Err: Estrogen-related receptor
FAOMeT: Farnesoic acid-O-methyl transferase
FDR: False discovery rate
Fem-1: Feminization-1
Fgf9: Fibroblast growth factor 9
Foxl2: Forkhead box L2
Fru: Fruitless
Gnrhr: Gonadotropin-releasing hormone receptor
GO: Gene ontology
H1D: Histone H1D
H2A: Histone H2A
Hpgds: Hematopoietic prostaglandin D synthase
Hsc70: Heat shock cognate 70
Hsp90: Heat shock protein 90
Iag: Insulin-like androgenic factor
KIFC1: Kinesin-like protein KIFC1
MF: Methyl farnesoate
Mih: Molt inhibiting hormone
Msl3: Male-specific lethal 3
Mylk: Myosin light chain kinase
NCBI: National Center for Biotechnology Information
Npc2: NPC intracellular cholesterol transporter 2 homolog a
nr: NCBI non-redundant and nucleotide database
ORF: Open Reading Frame
PE: Paired-end
Pges2: Prostaglandin E synthase 2
Pgmrc1: Progestin membrane receptor component 1
Ptgr1: Prostaglandin reductase 1
Pxt: Chorion peroxidase
Rspo1: R-spondin-1
Sf-1: Steroidogenic factor 1
Smad4: Mothers against decapentaplegic 4
Sox: Sry-related HMG box
SRA: Sequence Read Archive
Sry: Sex-determining region Y
Sxl: Sex-lethal
TnxB: Tenascin-X
TPM: Transcript Per Million
Tra2: Transformer 2
Vg: Vitellogenin
VgR: Vitellogenin receptor
Vmo1: Vitelline membrane outer layer protein 1
Wnt4: Wingless-type MMTV integration site family, member 4
