# Supplementary figures and images for: De novo gonad transcriptome analysis of the common littoral shrimp Palaemon serratus: novel insights into sex-related genes
Source: BMC Genomics. 2019 Oct 22;20:757. doi: 10.1186/s12864-019-6157-4 (PMC6805652; doi:10.1186/s12864-019-6157-4)

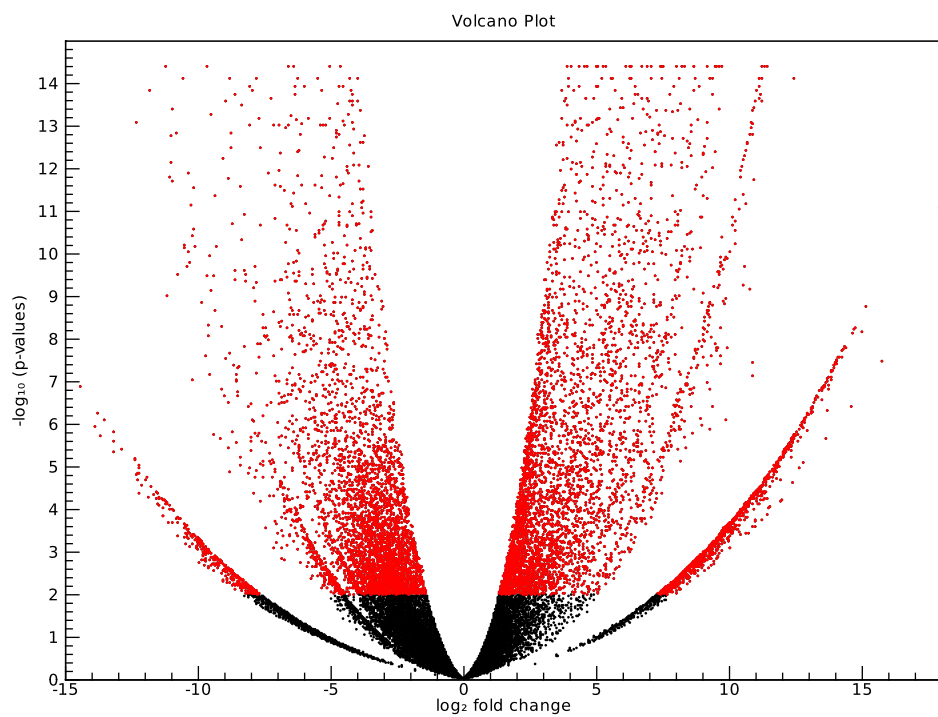

Supplement: Supplementary file 2 — Additional file 2: Figure S1. Volcano plot of differentially expressed genes (DEGs) between ovary and testis samples. Not differentially expressed genes are shown with black dots meanwhile DEGs are depicted with red dots. [file 12864_2019_6157_MOESM2_ESM.pdf]
